# Supplementary material for: ShrinkCRISPR: a flexible method for differential fitness analysis of CRISPR-Cas9 screen data
Source: BMC Bioinformatics. 2023 Feb 3;24:36. doi: 10.1186/s12859-023-05142-1 (PMC9896759; doi:10.1186/s12859-023-05142-1)
Supplement: Supplementary file 1 — Additional file 1. [file 12859_2023_5142_MOESM1_ESM.pdf]

Supplementary File for ShrinkCRISPR: A flexible method for differential fitness analysis of CRISPR-Cas9 screen data

Renaud Tissier, Janne van Schie, Rob Wolthuis, Job de Lange, Renée de Menezes

In this document, we present more detailed simulation results as well as additional information on the experimental data analysis.

Contents

A Simulations Results **2**

B Experimental Analysis **7**

B.1 Cisplatin sensitivity (paired design) 7

B.2 Longitudinal analysis (independent design) 7

A Simulations Results

A simulation study was set up to compare the performances of our proposed method with the different appraoches MAGeCK and drugZ in various settings. The different approaches were compared in terms of false positives and true positives genes detected in the simulated datasets. The simulations studies are presented in the section 3.1 of the paper. Figure S1 represents the distribution of the simulated lethality scores for the control cell type. Tables S1 and Table S2 displays the precision, recall and accuracy results of all scenarios for the independent design and the paired design, respectively. Table S3 shows the False positive and False negative simulations results for all scenarios. The reduced variance column shows the results obtained when reducing the variance of the sgRNA abundance at baseline between cell lines for an independent design screen. Figure S2 and Figure S3 display the same results in Figures 2 and 4 of the main text, respectively, here using boxplots. Table S4 shows the numbers of false positives obtained by shrinkCRISPR when the standard deviation σ of the distibution of fold changes is increased from 0.1 to either 0.5 or 1.

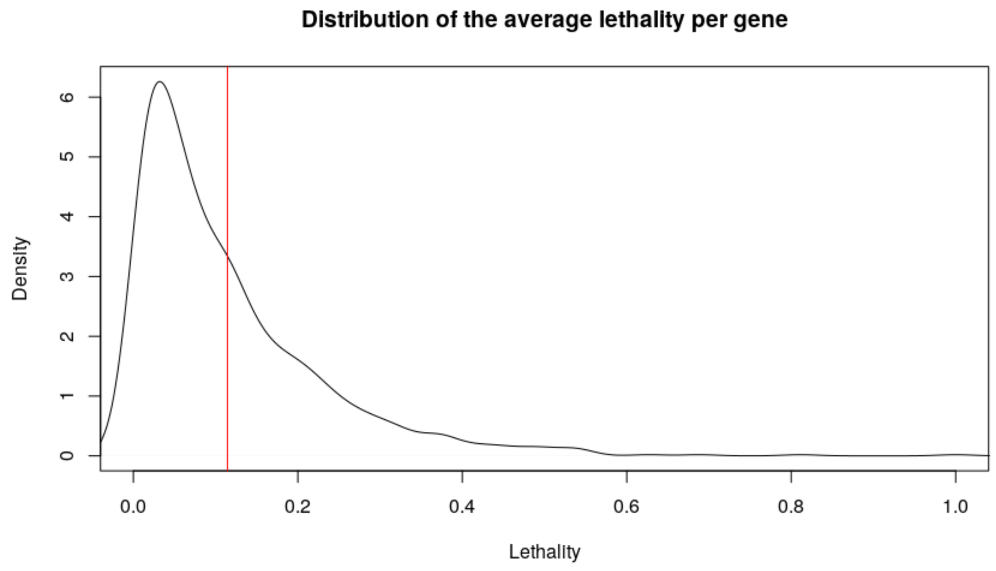

Figure S1: Density plot of the simulated average lethality scores from the 1000 simulated genes in a control cell replicate. The red horizontal line represent the mean of the distribution.

Table S1: Average precision, recall and accuracy results for each simulation scenario over 100 simulated datasets for the independent design. Into brackets are the standard deviations across datasets for each scenario. Missing values for the recall results indicates the absence of significant results for the methodology used.

	shrinkCRISPR			DrugZ			MagCEK		
Δ_g	Precision	Recall	Accuracy	Precision	Recall	Accuracy	Precision	Recall	Accuracy
0.1	0(0)	NA	0.90(0)	0.44(0.08)	0.63(0.07)	0.91(0.01)	0.20(0.03)	0.20(0.03)	0.83(0.01)
0.2	0.09(0.04)	1(0)	0.90(0.004)	0.80(0.10)	0.94(0.02)	0.97(0.01)	0.34(0.03)	0.33(0.03)	0.86(0.01)
0.3	0.83(0.05)	1(0)	0.98(0.005)	0.90(0.07)	0.98(0.01)	0.98(0.01)	0.37(0.03)	0.36(0.03)	0.87(0.01)
0.4	0.99(0.01)	1(0)	0.99(0.001)	0.92(0.07)	0.98(0.01)	0.99(0.01)	0.34(0.04)	0.35(0.04)	0.87(0.01)
0.5	0.99(0.01)	1(0)	0.99(0.001)	0.90(0.07)	0.99(0.01)	0.08(0.01)	0.31(0.04)	0.33(0.04)	0.86(0.01)

Table S2: Average precision, recall and accuracy results for each simulation scenario over 100 simulated datasets for the paired design. Into brackets are the standard deviations across datasets for each scenario. Missing values for the recall results indicates the absence of significant results for the methodology used.

	shrinkCRISPR			DrugZ			MagCEK		
Δ_g	Precision	Recall	Accuracy	Precision	Recall	Accuracy	Precision	Recall	Accuracy
0.1	0(0)	NA	0.90(0)	0.12(0.03)	0.12(0.03)	0.82(0.01)	0.10(0.02)	0.10(0.03)	0.82(0.01)
0.2	0.07(0.03)	1(0)	0.91(0.01)	0.19(0.03)	0.20(0.04)	0.84(0.01)	0.11(0.03)	0.11(0.03)	0.82(0.01)
0.3	0.79(0.05)	1(0)	0.97(0.01)	0.30(0.04)	0.36(0.06)	0.87(0.01)	0.15(0.03)	0.15(0.03)	0.83(0.01)
0.4	0.99(0.01)	1(0)	0.99(0.001)	0.42(0.04)	0.58(0.08)	0.91(0.01)	0.20(0.03)	0.20(0.03)	0.84(0.01)
0.5	0.99(0.01)	1(0)	0.99(0.001)	0.52(0.05)	0.82(0.08)	0.94(0.01)	0.29(0.03)	0.29(0.04)	0.86(0.01)

Table S3: Average number of false positive hits (FP) and true positive hits (TP) for each simulation scenario. Into brackets are the standard deviations across datasets for each scenario.

Method	Δ_g	Paired Design		Independent Design		Reduced variance	
		TP	FP	TP	FP	TP	FP
	0	-	0(0)	-	0(0)	-	-
	0.1	0(0)	0(0)	0(0)	0(0)	-	-
ShrinkCRISPR	0.2	8.1(4.7)	0(0)	7.0(3.9)	0(0)	-	-
	0.3	81.7(6.9)	0(0)	78.0(7.4)	0(0)	80.1(6.2)	0(0)
	0.4	99.6(0.8)	0(0)	99.1(0.6)	0(0)	-	-
	0.5	99.9(0.4)	0(0)	99.8(0.5)	0(0)	-	-
	0	-	105.2(18.9)	-	101.4(6.7)	-	-
	0.1	20.7(7.5)	85.1(8.0)	11.3(3.7)	89.7(6.3)	-	-
MaGCEK	0.2	33.3(8.9)	71.4(2.1)	12.8(4.3)	89.3(7.6)	-	-
	0.3	35.8(7.1)	67.2(1.3)	17.1(4.1)	85.7(12.6)	32.5(5.0)	71.4(6.6)
	0.4	33.0(6.7)	65.6(0.4)	22.7(3.6)	79.9(12.2)	-	-
	0.5	29.6(9.4)	65.6(0.5)	33.1(4.9)	70.9(7.3)	-	-
	0	-	66.9(6.1)	-	101.3(7.0)	-	-
	0.1	46.8(3.3)	30.5(6.1)	13.3(3.2)	86.3(7.0)	-	-
drugZ	0.2	81.2(2.5)	6.1(5.9)	21.8(2.8)	75.3(7.2)	-	-
	0.3	90.2(3.5)	2.1(7.0)	31.3(3.4)	54.1(6.7)	61.8(3.2)	12.8(6.6)
	0.4	89.9(3.8)	1.5(5.6)	42.9(4.5)	30.2(6.29)	-	-
	0.5	89.4(3.7)	0.7(5.7)	55.1(5.6)	11.7(5.9)	-	-

Table S4: Average number of false positive hits (FP) for each simulation scenario with the standard deviation of the fold changes equal to 0.5 and 1. Into brackets are the standard deviations across datasets for each scenario.

Δ_g	$\sigma = 0.5$	$\sigma = 1$
0	2.3(1.7)	5.9(1.6)
0.1	3.0(1.8)	6.0(1.8)
0.2	3.7(2.1)	6.5(2.3)
0.3	4.8(2.3)	6.6(1.9)
0.4	7.6(3.6)	7.3(2.7)
0.5	12.9(3.8)	8.1(3.3)

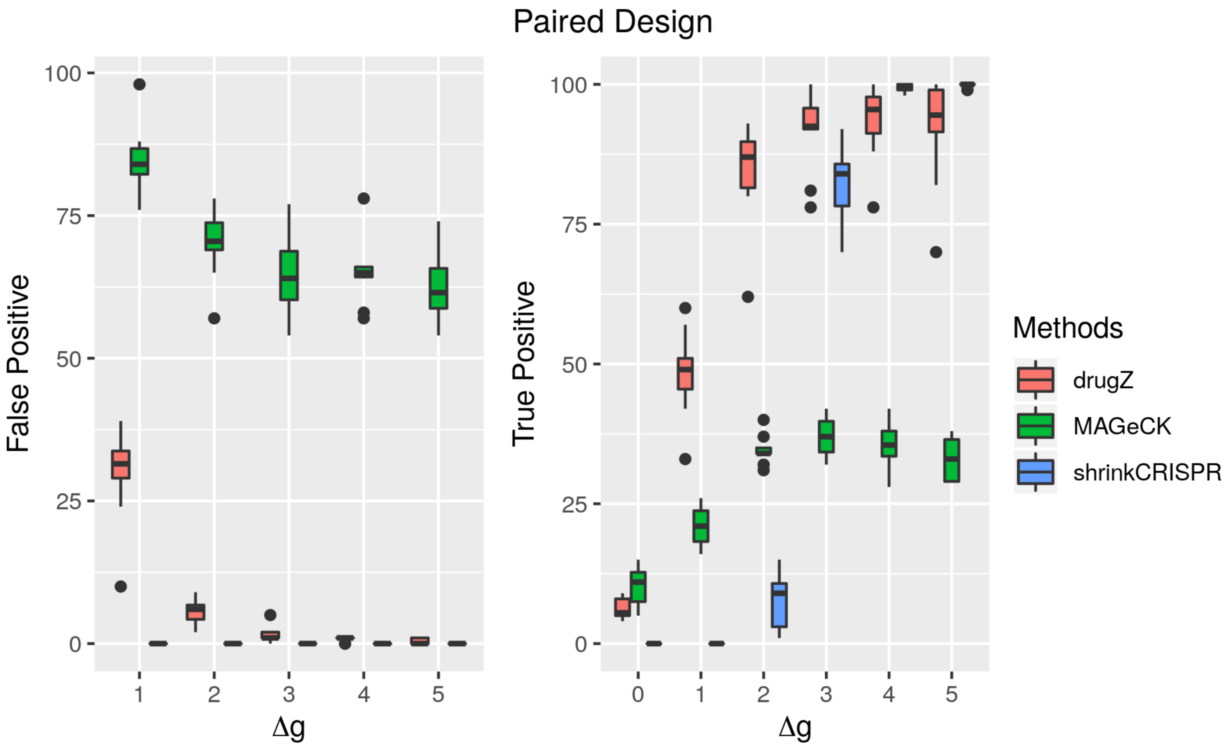

Figure S2: Results per method and simulation scenario, for the paired design. Left panel: average number of false positive hits. Right panel: average number of true positive hits. Standard deviation of the number of true and false positives across simulated datasets are represented by the black lines.

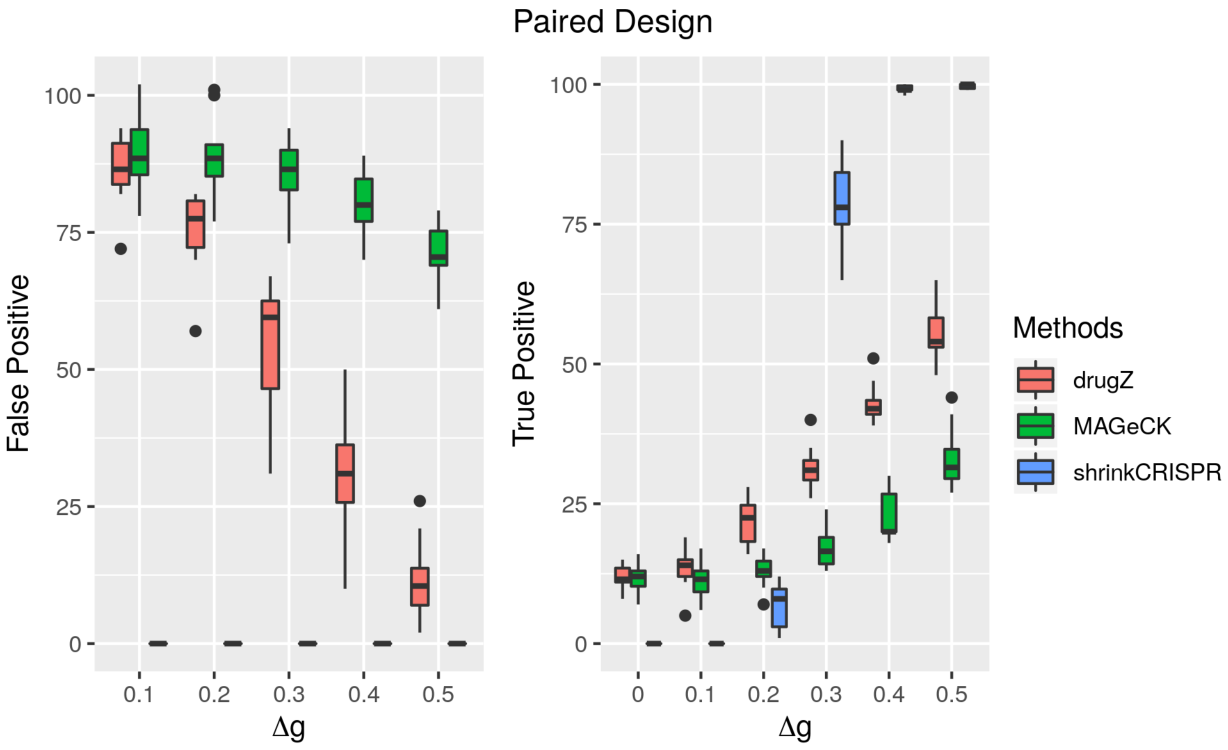

Figure S3: Results per method and simulation scenario, for the paired design. Left panel: average number of false positive hits. Right panel: average number of true positive hits. Standard deviation of the number of true and false positives across simulated datasets are represented by the black lines.

B Experimental Analysis

B.1 Cisplatin sensitivity (paired design)

Using shrinkCRISPR, we analysed cisplatin sensitivity screens data, which were part of a large study of the sensitivity to a variety of drugs of human RPE1 cells (see main text). From this analysis, 37 genes were found significant. Figure S4 displays the fitness scores obtained by ShrinkCRISPR for the 37 genes in the two conditions: treated with cisplatin and without cisplatin.

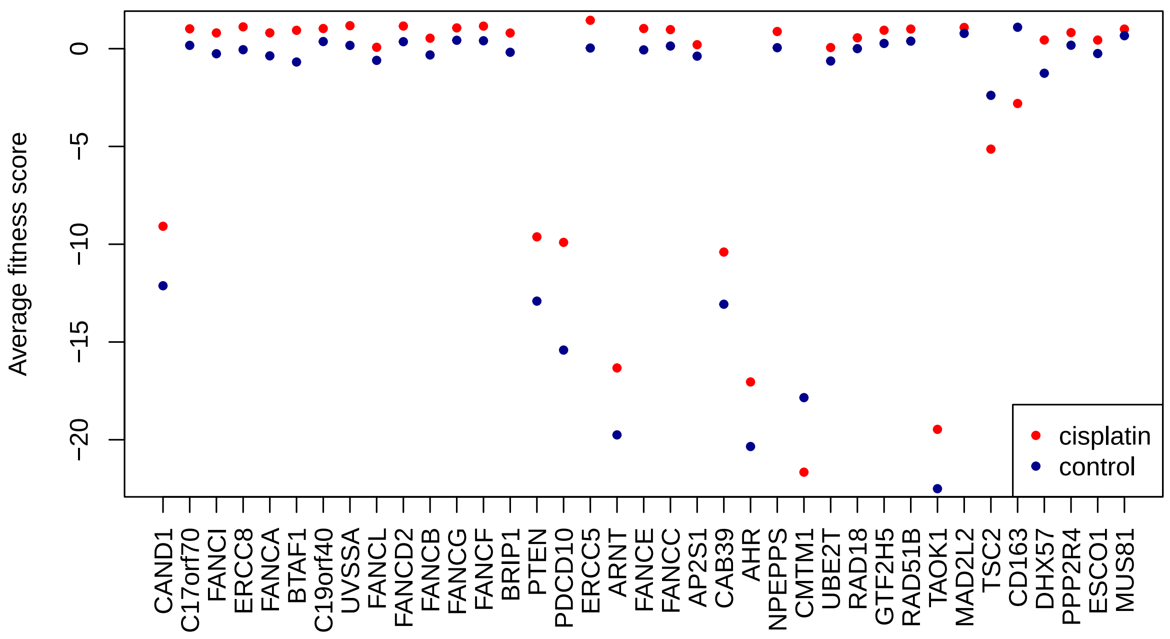

Figure S4: Average fitness score for both cisplatin treated cells (red) and control cells (blue). A positive fitness score indicates depletion of the sgRNA over time; negative values indicate enrichment.

B.2 Longitudinal analysis (independent design)

We used ShrinkCRISPR to analyse the publicly available data for screens produced by the Moffat lab (http://tko.ccbr.utoronto.ca) and previously analysed in [1]. Six differents cell lines (DLD1, GBM, HCT116_1, HCT116_2, HeLa and RPE1) have been screened at several time points. Each cell line was screened in duplicate or triplicate. A summary of the number of samples available at each time point per cell line is presented in table S5. Figure S5 illustrates the intersection of the significant genes obtained by ShrinkCRISPR

for each individual time point.

Table S5: Number of replicates at each measured time point per cell line in the TKO library publicly available dataset.

Time Point	DLD1	GBM	HCT116_1	**HCT116_2**	**HeLa**	RPE1
$T = 0$	1	1	1	**1**	**1**	1
$T = 1$	1	2	2	**3**	**3**	2
$T = 2$	1	2	2	**3**	**3**	2
$T = 3$	1	2	2	**3**	**3**	2
$T = 4$	0	0	2	**3**	**3**	2
$T = 5$	0	0	2	**0**	**0**	0

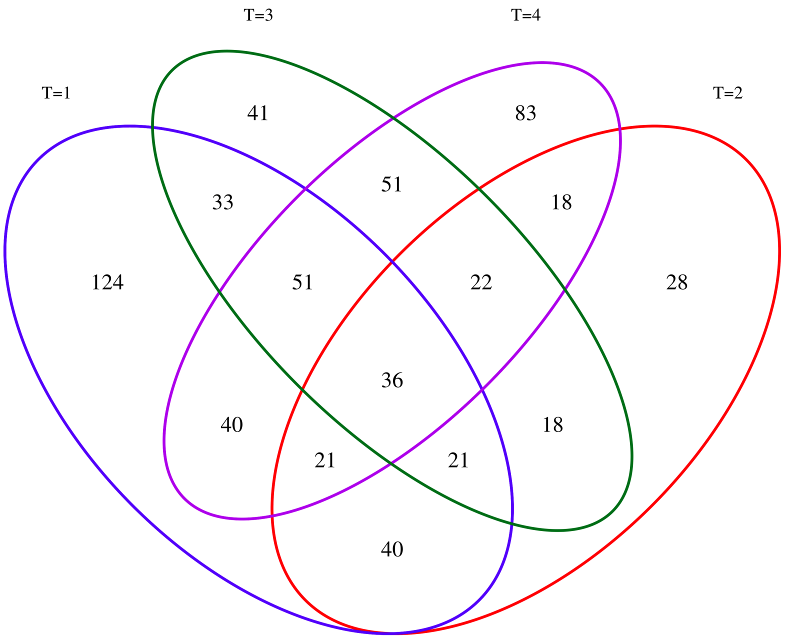

Figure S5: Venn diagram illustrating the overlap between the genes presenting a differential effect on cell fitness between the HeLa and HCT116_2 cell lines for the 4 analyses ran at the 4 different time points.

References

[1] T. Hart, M. Chandrashekhar, M. Aregger, Z. Steinhart, K.R. Brown, G. MacLeod, M. Mis, M. Zimmermann, A. Fradet-Turcotte, S. Sun, P. Mero, P. Dirks, S. Sidhu, P. Roth, O.S. Rissland, D. Durocher, S. Angers, and J. Moffat. High-resolution crispr screens reveal fitness genes and genotype-specific cancer liabilities. *Cell*, 163(6):1515–26, 2015.
